# Prevalence of Cardiovascular Risk Factors in Saudi Patients With Psoriatic Arthritis: A Single-Center Retrospective Cohort Study

**DOI:** 10.7759/cureus.46570

**Published:** 2023-10-06

**Authors:** Mishari T Alrubaiaan, Saad A Alsulaiman, Abdullah N Altasan, Abdullah Alqahtani, Abdulrahman Alrashid, Osama L Mohamed

**Affiliations:** 1 Medicine, King Saud Bin Abdulaziz University for Health Sciences, Riyadh, SAU; 2 Rheumatology, King Abdulaziz Medical City Riyadh, Riyadh, SAU; 3 Biostatistics, King Abdullah Biography Center, Riyadh, SAU

**Keywords:** psa, cvd, cardiovascular risk factors, psoriasis, psoriatic arthritis

## Abstract

Background

Psoriatic arthritis (PsA) is an extremely heterogeneous disease with numerous articular phenotypes and extra-articular manifestations. It is common for patients with PsA to have coexisting medical conditions. In recent studies, PsA patients were found to have a greater prevalence of cardiovascular risk factors when compared to non-PsA groups.

Objectives

This study aimed to describe the prevalence of cardiovascular risk factors among Saudi psoriatic arthritis patients treated at King Abdulaziz Medical City (KAMC), Riyadh.

Methods

A hundred and twenty-six patients with psoriatic arthritis diagnoses were enrolled in this study. Patients who were 18-years-old or older, had PsA diagnosed by a rheumatologist, and met the Classification Criteria for Psoriatic Arthritis (CASPAR) criteria were included in the study population. Patients were excluded from the study if they were younger than 18, did not fulfill the CASPAR criteria, did not have a documented diagnosis by a rheumatologist, or had been diagnosed with any type of joint arthritis in the past. In this retrospective cohort article, we investigated the frequency of risk factors for cardiovascular disease such as [hypertension (HTN), dyslipidemia (DLP), diabetes mellitus (DM), obesity, and coronary heart disease (CHD)] and non-established risk factors such as [HbA1C, erythrocyte sedimentation rate (ESR) and C-reactive protein (CRP)]. SPSS version 12.0 for Windows (SPSS Inc.) was used for statistical analysis. The threshold for statistical significance was set at 5%.

Results

A hundred and twenty-six PsA patients were enrolled in this study, 30 (24%) had PsA for less than two years (early), and 96 (76%) had PsA for more than two years (established). When the analysis was performed, the mean age was 47.5 years, and the mean age at diagnosis of PsA was 42.4 years. Of them, 89 (71%) were female while 37 (29%) were male. Established PsA patients were significantly older at the time of analysis than early PsA patients (49.2 vs. 41.8 years; P= 0.007). Furthermore, established PsA patients had a longer duration of PsA than those with early PsA (6.3 vs. 1.5 years; P= <0.001). The most frequently reported comorbidity was obesity (61%) followed by DLP (43%), HTN (34%), DM (30%), and CHD (11%). CV comorbidities did not differ between subgroups. However, patients with established PsA had a higher prevalence of DLP, especially females. Additionally, patients with early PsA had greater rates of HTN than those with established PsA, and patients with early PsA were more likely to have CHD.

Conclusion

This study confirms that PsA is linked with cardiovascular (CV) morbidity. When evaluating PsA, future studies should take these CV conditions into consideration.

## Introduction

Psoriatic arthritis (PsA) is an inflammatory musculoskeletal disorder related to psoriasis (PsO) [1-3]. Even though 15% of patients experience PsA concurrently with PsO, it usually occurs 10 years before PsA [1,2,4]. Moreover, the prevalence of PsA is estimated to range from 10% to 40% in patients with PsO [1,2,4]. There are several symptoms of PsA, including peripheral arthritis, central arthritis, enthesitis, dactylitis, and occasionally nail dystrophy [4-6]. When it comes to diagnosing and classifying PsA, the Classification Criteria for Psoriatic Arthritis (CASPAR) is usually used [7].

Patients with PsO are more likely to suffer from premature CVD [4]. According to recent studies, patients with PsO have a higher tendency to have CV risk factors, such as hypertension (HTN), dyslipidemia (DLP), diabetes mellitus (DM), and obesity [3,4,8,9]. So far, the studies that have investigated the prevalence of CV risk factors among patients with PsA reported that PsA patients were most likely to die from circulatory diseases as well [10]. Furthermore, according to Gladman et al., patients with PsA were more likely to suffer from myocardial infarction, angina, and hypertension [11]. In addition, according to Khraishi et al., PsA had a significantly higher rate of hypertension than the general population [12]. Similarly, the prevalence of HTN among PsA patients was recently found to be higher than that reported among PsO patients without arthritis [13]. To date, the CV risk factors in Saudi patients with PsA are not well understood. Therefore, a lack of accurate detection may result in delayed interventions in clinical practice. Hence, further research is needed to investigate the potential link between PsA and CVD risk.

Our study aimed to investigate the frequency of cardiovascular (CV) risk factors, such as HTN, DLP, DM, obesity, and CHD, and non-established risk factors such as [HbA1C, erythrocyte sedimentation rate (ESR) and C-reactive protein (CRP)] among PsA cases in Saudi Arabia attending the rheumatology department at King Abdulaziz Medical City (KAMC), Riyadh. We hypothesized that a prolonged period of inflammation in PsA cases has an increased frequency of CV risk factors.

## Materials and methods

Study design

A single-center retrospective cohort study of subjects with PsA documented in the BESTCare system from 2015 to 2023 attending the rheumatology department at KAMC. KAMC is a tertiary care center that started operating in May 1983, and since then, it has been distinguished as an important healthcare center in the area. Using the BESTCare System nearly all clinically recognized cases of PsA can be captured, along with access to complete medical records for data collection. This study design was cost-effective and safe. It did not interfere with patients’ treatment plans. No extra investigations were carried out other than those that were already performed as part of the standard medical care provided to the patients.

Selection criteria

The study population consisted of patients aged 18 or older with a PsA diagnosis documented by a rheumatologist and who have fulfilled the CASPAR criteria. Patients were excluded from the study if they were younger than 18, did not fulfill the CASPAR criteria, did not have a documented diagnosis by a rheumatologist, or had been diagnosed with any type of joint arthritis in the past.

Sample size

The total number of PsA cases in KAMC is 168 cases from 2015 to 2023. After meeting the inclusion and exclusion criteria, our sample size was 126 patients with PsA.

Data collection

The study group identified and reviewed the complete medical records of all clinically recognized subjects with PsA using a standardized data abstraction form. Two subgroups of PsA patients were defined. Patients with early PsA, defined as those diagnosed within the last two years, were included in the first subgroup, while the second subgroup included patients with established PsA, defined as those with two or more years of diagnosis [3].

The frequency of multiple CV risk factors was examined. Medical records were reviewed for comorbidities recorded by the physicians. The smoking history was presented as (current or never). HTN was determined as at least two blood pressure measurements over 140mmHg systolic and 90mmHg diastolic, has been diagnosed with HTN by a physician, or is on medications for hypertension [14]. DM was determined as at least two fasting plasma glucose measurements of 126mg/dl, two-hour plasma glucose levels of 200mg/dl, or taking medications for DM [14]. In terms of DLP, it was determined as having a total cholesterol (TC) level of ≥5.2 mmol/L or the use of medications that lower cholesterol [14]. Those with a body mass index (BMI) greater than 30kg/m2 were considered obese [14]. Patients were considered to have CHD if they had a current or previous history of myocardial infarction, angina, and transient ischemic attack [3]. Aside from evaluating the established risk factors such as age, gender, smoking, HTN, diabetes, obesity, DLP, and CHD, non-established risk factors including HbA1C, ESR, and CRP were also evaluated.

Statistical analysis

For continuous variables, descriptive statistics such as the mean and standard deviation (SD) were used, and frequency distributions for discrete variables were reported. For the analysis of differences between subgroups, Chi-square and Fisher exact tests were used. The threshold for statistical significance was set at 5%, and all tests were two-tailed. For all statistical analyses, SPSS version 12.0 for Windows (SPSS Inc., Chicago) was used.

Ethical consideration

The study received its ethical approval and Institutional Review Board (IRB) approval from King Abdullah International Medical Research Center (KAIMRC) with the reference number: NRC23R/304/05.

## Results

A hundred and twenty-six PsA patients were enrolled in this study, 30 (24%) had PsA for less than two years (early), and 96 (76%) had PsA for more than two years (established). The demographic and baseline characteristics are summarized in Table 1. When the analysis was performed, the mean age was 47.5 years, and the mean age at diagnosis of PsA was 42.4 years. A total of 89 (71%) of the patients were female with a mean age of 48.4 while 37 (29%) were male with a mean age of 45.2. In terms of inflammatory markers, the mean ESR (available in 121 patients) was 36.1 mm/hr (SD: 25.4), and the mean CRP (available in 110 patients) was 9.5 mm/hr (SD: 19.1). In terms of demographics, there were no major variations between the PsA subgroups. However, when the study was conducted, patients with PsA for more than two years (established) were older than patients with PsA for less than two years (early) (49.2 vs. 41.8 years; P= 0.007). Furthermore, in established PsA cases, the mean PsA duration was 6.3 years, while in early PsA cases, the mean PsA duration was 1.5 years (6.3 vs 1.5 years; P= <0.001).

**Table 1:** The demographics and baseline characteristics of the total patients and patients in the PsA cohorts N: number; SD: standard deviation PsA: psoriatic arthritis; ESR: erythrocyte sedimentation rate; CRP: c-reactive protein

Characteristics	"	Early	Established	All	P	Sig
Number of Patients N(%)		30(24%)	96(76%)	126(100%)		
Gender N(%)	Male	3(10%)	34(35%)	37(29%)	0.008	*
	Female	27(90%)	62(65%)	89(71%)	0.008	*
Age Mean(SD)	All	41.8(12.5)	49.2(13.2)	47.5(13.4)	0.007	*
	Male	42 (20.6)	45.5 (15.8)	45 (15.9)	0.717	
	Female	41.7 (11.8)	51.3 (11.1)	48.4 (12.1)	<0.001	*
Age at diagnosis of PsA Mean(SD)		40.4(12.7)	43.0(13.3)	42.4(13.2)	0.353	
Duration (year) of PsA Mean(SD)		1.5(0.5)	6.3(2.8)	5.1(3.2)	<0.001	*
ESR Mean(SD)		37.6(23.7)	35.7(26.0)	36.1(25.4)	0.733	
CRP Mean(SD)		12.3(18.1)	8.7(19.4)	9.5(19.1)	0.399	

The data presented in Table 2 details the CV risk factors overall and by PsA subgroups. (4 %) of the patients were smokers and (96 %) were non-smokers. The mean body mass index (BMI) was 31.9 kg/m2 (SD: 6.5), and 61% were obese, defined as having a BMI of 30 or over. Among the patients, obesity (61%) was the most prevalent comorbidity, followed by DLP (43%), hypertension (34%), diabetes (30%), and CHD (11%). The mean systolic blood pressure was 128 mmHg (SD: 14.8) and the mean diastolic blood pressure was 72.1 mmHg (SD: 11.1). The mean HbA1C level (available in 85 patients) was 6.5 (SD: 1.5) while the mean HbA1C in diabetic patients with PsA was 7.7. The lipid levels (available in 98 patients) consisted of; mean total cholesterol which was 4.6 mmol/L (SD: 1.1); mean high-density lipoprotein (HDL) level which was 1.3 mmol/L (SD: 0.3); mean low-density lipoprotein (LDL) which was 2.9 mmol/L (SD: 1.0); and mean triglyceride which was 1.3 mmol/L (SD: 0.7). There was a higher prevalence of obesity among female patients than male patients. Similarly, the prevalence of DLP was higher in female patients compared to males. Additionally, compared to male patients, female patients had a higher prevalence of DM. When it came to the prevalence of CV comorbidities between PsA subgroups, it was relatively similar. However, DLP was more prevalent in established PsA cases, especially in females, while early PsA cases were more likely to have HTN, especially in males, and CHD. Despite this, we did not find any major differences between PsA subgroups.

**Table 2:** Cardiovascular risk factors and comorbidity profiles overall and by PsA cohorts stratified by duration of PsA and gender. N: number; SD: standard deviation BMI: body mass index; HbA1C: glycated hemoglobin; SBP: systolic blood pressure; DBP: diastolic blood pressure; HDL: high-density lipoprotein; LDL: low-density lipoprotein

		Male			Female			Total		
		EARLY	ESTABLISHED	TOTAL	EARLY	ESTABLISHED	TOTAL	EARLY	ESTABLISHED	TOTAL
Smoking N(%)	Yes	0(0%)	5(15%)	5(14%)	0(0%)	0(0%)	0(0%)	0(0%)	5(5%)	5(4%)
	No	3(100%)	29(85%)	32(87%)	27(100%)	62(100%)	89(100%)	30(100%)	91(95%)	121(96%)
Obesity N(%)	Yes (BMI>30)	2(67%)	17(50%)	19(51%)	17(63%)	41(66%)	58(65%)	19(63%)	58(60%)	77(61%)
	No (BMI<30)	1(33%)	17(50%)	18(49%)	10(37%)	21(34%)	31(35%)	11(37%)	38(40%)	49(39%)
BMI Mean(SD)		34.8(6.2)	30.1(7.3)	30.5(7.3)	31.6(5.6)	32.8(6.2)	32.5(6.0)	31.9(5.6)	31.9(6.7)	31.9(6.5)
Diabetes mellitus N(%)	Yes	1(33%)	7(21%)	8(22%)	8(30%)	22(36%)	30(34%)	9(30%)	29(30%)	38(30%)
	No	2(67%)	27(79%)	29(78%)	19(70%)	40(65%)	59(66%)	21(70%)	67(70%)	88(70%)
HbA1C Mean(SD)		5.6(0.4)	6.4(1.7)	6.3(1.6)	6.8(1.8)	6.4(1.3)	6.5(1.4)	6.6(1.7)	6.4(1.4)	6.5(1.5)
HbA1C of diabetic patients only Mean(SD)		5.8 (only 1 data point)	8.7(1.4)	8.3(1.7)	8.1(1.6)	7.4(1.3)	7.6(1.4)	7.9(1.7)	7.7(1.4)	7.7(1.5)
Hypertension N(%)	Yes	2(67%)	11(32%)	13(35%)	9(33%)	21(34%)	30(34%)	11(37%)	32(33%)	43(34%)
	No	1(33%)	23(68%)	24(65%)	18(67%)	41(66%)	59(66%)	19(63%)	64(67%)	83(66%)
SBP Mean(SD)		123.7(23.0)	129.4(15.6)	128.9(16.0)	124.5(14.0)	128.9(14.5)	127.6(14.4)	124.4(14.6)	129.1(14.8)	128.0(14.8)
DBP Mean(SD)		73.3(11.1)	75.5(7.5)	75.3(7.6)	71.3(10.9)	70.5(12.5)	70.7(12.0)	71.5(10.8)	72.2(11.2)	72.1(11.1)
Dyslipidemia N(%)	Yes	1(33%)	14(41%)	15(41%)	9(35%)	29(48%)	38(44%)	10(35%)	43(45%)	53(43%)
	No	2(67%)	20(59%)	22(60%)	17(65%)	32(53%)	49(56%)	19(66%)	52(55%)	71(57%)
Total cholesterol Mean (SD)		0 (0)	4.4(1.2)	4.4(1.2)	4.7(0.9)	4.6(1.2)	4.7(1.1)	4.7(0.9)	4.6(1.2)	4.6(1.1)
HDL Mean (SD)		0 (0)	1.1(0.3)	1.1(0.3)	1.4(0.3)	1.3(0.2)	1.3(0.3)	1.4(0.3)	1.2(0.3)	1.3(0.3)
LDL Mean (SD)		0 (0)	2.7(1.2)	2.7(1.2)	3.0(0.8)	3.0(1.0)	3.0(1.0)	3.0(0.8)	2.9(1.1)	2.9(1.0)
Triglycerides Mean (SD)		0 (0)	1.4(0.9)	1.4(0.9)	1.2(0.7)	1.2(0.5)	1.2(0.6)	1.2(0.7)	1.3(0.7)	1.3(0.7)
Coronary heart disease (CHD) N (%)	Yes	1(33%)	2(6%)	3(8%)	5(19%)	6(10%)	11(12%)	6(20%)	8(8%)	14(11%)
	No	2(67%)	32(94%)	34(92%)	22(82%)	56(90%)	78(88%)	24(80%)	88(92%)	112(89%)

## Discussion

According to previous studies, patients with PsA have a higher prevalence of CV risk factors than control individuals or those with PsO. In the current study, we examined the prevalence of CV risk factors among PsA patients at KAMC, Riyadh. Additionally, to examine the potential influence of inflammation on CV factors, a subgroup analysis was performed. Obesity was the most frequently reported comorbidity (61% of patients), followed by DLP (43%), HTN (34%), DM (30%), and CHD (11%).

Compared to the general Saudi population, the presence of CVD risk factors was much higher in our PsA group. For instance, the maximum reported obesity prevalence in the Saudi population was 35% in adults over 18 years old, while the prevalence of cigarette smoking was 14% [15,16]. Within our PsA group, 61% were classified as obese, while only 4% were smokers. Moreover, the prevalence of DLP was found to be 54% among the general Saudi population which is quite comparable to our finding in the PsA group 43% [17].

When it comes to comparing our results with previous studies that have addressed the CV profile of patients with PsA, obesity emerged as the most common comorbidity among patients with PsA. In total, 77 patients were obese, equating to a frequency of 61% which is much higher than the estimated frequency in prior studies [8,11,13]. In addition, our patients had a much higher frequency of DLP 43% [8,11,13]. Khraishi et al. suggested that regional differences in nutrition or a different definition of DLP may explain the similar prevalence of DLP and obesity in their study [3]. Despite this, HTN ranked third in our study among comorbidities, affecting 34% of patients. It was consistent with previously reported PsA studies where HTN ranged from 25% to 49% [8,11,13]. However, it was higher than what was reported in the Saudi general population 26% [18]. Moreover, Gladman et al., found that HTN prevalence in patients with PsA was significantly higher compared with that in their PsO group without arthritis [13]. Furthermore, according to recent studies, type 2 DM prevalence ranges from 6% to 20% in PsA patients, which is generally lower than the prevalence of DM in our PsA patients which was 30% [3,5,8,13]. Increased levels of adipokines may contribute to the relationship between PsA and DM, according to Dal Bello et al. [4].

Moreover, for a patient sample with a similar age (mean age 48.98), Khraishi et al. reported a similar prevalence of CHD [3]. CV risk factors were similar across subgroups. However, similar to Khraishi et al., females with established PsA had higher DLP [3]. Furthermore, our findings showed that HTN prevalence was higher in males with early PsA while in Khraishi et al., study it was slightly higher among male patients with established PsA [3]. Finally, compared to patients with established PsA, patients in both genders with early PsA had a higher prevalence of CHD which corresponds with Khraishi et al. [3].

It is important to consider several limitations in this study when interpreting its findings. First, this is a retrospective cohort study, conducted without controlled experiments, based on medical records. Second, This study involved only 126 PsA patients from a single center, so generalizing to other groups may be difficult. In order to accurately estimate the prevalence of CV risk factors in patients with PsA, future studies will require a larger sample size of patients with PsA. Moreover, since this is a prevalence study, it is not possible to determine whether comorbid conditions (DLP, HTN, DM, obesity, and CHD) preceded or followed PsA.

## Conclusions

This study aimed to evaluate the prevalence of CV risk factors among PsA patients and to correlate the length of PsA symptoms to these risk factors. The data, as a whole, offers some interesting evidence in favor of PsA's influence on CV morbidity. According to the data, obesity (61%) was the most prevalent comorbid condition, followed by DLP (43%) as the second comorbid disease, followed by HTN (34%), DM (30%), and CHD (11%).
